# The interpretation of discrepancies between peer victimization experiences reported by different informants in capturing victimization‐related genetic liability. A commentary on Armitage et al. (2022)

**DOI:** 10.1002/jcv2.12137

**Published:** 2023-01-20

**Authors:** Annalisa Lella, Linda A. Antonucci, Giulio Pergola

**Affiliations:** ^1^ Department of Translational Biomedicine and Neuroscience University of Bari Aldo Moro Bari Italy

**Keywords:** gene‐environment correlations, peer victimization, polygenic scores

## Abstract

A recent work published in this journal by Armitage et al. reported that wellbeing‐related genetic scores (PGS) are associated with self‐informed peer victimization questionnaires. In contrast, peer‐ and teacher‐informed measures would capture intelligence and educational attainment PGS better. However, we argue that this dichotomy does not find comprehensive support in the literature; instead, informants other than self and especially peers may provide reports from angles particularly relevant to mental health. For example, peer reports may more objectively capture adverse social reactions evoked by genetic factors (i.e., evocative gene‐environment correlations). Thus, we recommend caution in generalizing the conclusion that self‐reports capture the association between genetic contribution to mental health and peer victimization better than other‐informant measures, as different gene‐environment mechanisms may be at play.

Psychiatric disorders result from a complex interplay between genetic vulnerability and environmental factors in such a tight relationship that vulnerability could be modeled as a genetic susceptibility to environmental risk (Weinberger, 2019). Characterizing such gene‐environment interplays is expected to help tailor interventions to promote mental health and prevent psychiatric conditions. The interplay between genetic and environmental factors may be categorized into different types.

Gene‐environment interactions (G×E) are mechanisms in which genetic and environmental factors occur independently, but each factor's effect on a given phenotype is conditional upon the interacting factor. Instead, gene‐environment correlations (rGE) reflect the degree of covariance between genetic and environmental factors. Multiple types of rGE may be observed (Plomin, 1986). Parental influences transmitted to a child are a case of a passive rGE. For example, intelligent parents may not only provide their child with genes associated with high cognitive performance but also with environmental stimuli promoting high intelligence. Another rGE type is represented by evocative rGE, which reflect how genes modify environmental factors by eliciting environmental reactions, for example, social reactions. Active rGE reflect genetically influenced niche picking, like the selection of a deviant environment by someone with genetic factors associated with antisocial behavior. The rGE may explain the heritability increase over the lifespan of cognitive phenotypes such as intelligence, potentially reflecting the reinforcement of genetic tendencies by the environment. Characterizing GE interplays would allow to detect and prevent potentially detrimental effects of such interplays. For example, intervening on their environmental component is possible through training programs and policy making. In the current GWAS era, polygenic risk scores, summarizing the estimated effect of numerous genetic variants derived from GWAS analysis on an individual's phenotype, represent the workhorse of studies aimed to analyze multiple genetic factors in association with the effects of the environment.

In this context, Armitage et al. (2022) in a recent issue of *JCPP Advances* linked genetic risk factors in the form of polygenic scores (PGSs) related to mental health, cognitive abilities and physical traits with peer victimization—unmistakably an environmental factor as it does not directly depend on the gene carrier. The authors assessed peer victimization using longitudinal reports by multiple informants during childhood in 536 participants from the Quebec Newborn Twin Study (QNTS), and investigated whether reports from different informants could provide different insights on genetic liabilities related to this phenomenon. They found that the statistically significant associations between PGSs and victimization differed across informants. Specifically, self‐reported peer victimization was significantly associated with Wellbeing‐related PGS, teacher‐reported victimization with Intelligence‐related, body mass index (BMI)‐related and Educational Attainment‐related PGSs, and peer‐reported victimization with Educational Attainment‐related PGS. The authors claimed that reports made by different informants might potentially capture different genetic liabilities related to peer victimization and therefore encouraged embracing a multifaceted perspective—a viewpoint we concur with. We also commend the effort to address informant differences in characterizing gene‐environment interplay in a longitudinal cohort. On the other hand, we caution against interpreting potential measurement uncertainty as a biological phenomenon. For instance, the interpretation offered in the article that self‐reports may capture genetic risk for internalizing conditions, or more generally, mental well‐being, whereas peer‐ and teacher‐reports may capture maladjustment and/or more cognitive‐related traits, seems premature based on a genetic study of this sample size and difficult to reconcile with other published evidence.

When investigating the genetics of mental health traits in general, we need to consider that individuals at increased genetic risk may be more likely to report negative peer treatment (Plomin, 1986), as also Armitage et al. highlight. Reports from others are, likewise, influenced by their genetics, but this potential source of bias is conceivably curbed by gathering data from multiple individuals. On the other hand, specific forms of gene‐environment interplay like evocative rGE, which depend on reactions by other individuals to behaviors displayed by the gene carrier, are plausibly best assessed by other informants. In the case of evocative rGE, a genetic predisposition to traits like depression or paranoia in children may elicit peer reactions (e.g., peer victimization) aligned with genetic risk, hence resulting in correlated genetic and environmental pressures. As the carrier's genetics (e.g., risk for depression) do not directly influence the responses from other informants except through evocative rGE, but may, instead, influence the perception of victimization and thus affect self‐reports, the former are more reliable than the latter when addressing evocative rGE.

These arguments raise another question: are peer reports more reliable in measuring peer victimization without regard to genetics, compared with self‐ and teacher‐ reports? Regarding information about aggression in older children and adolescents, the literature backs this assumption (Clemans et al., 2014). However, self‐reports accurately identify maladaptive or poor wellbeing outcomes in late adolescence and early adulthood (Cornell & Cole, 2012). Nevertheless, even in these life stages, reports from other informants—both teachers and peers (Clemans et al., 2014)—could capture other aspects of mental health, for example, those more related to role functioning and social adjustment. These very aspects of behaviors could carry relevant information regarding psychiatric risk, therefore, the dichotomy outlined by the authors for which “*genetic predictors of peer victimization are influenced by the informant*, *with self‐reports more associated with genetic risk for mental health problems*, *and teacher‐ and peer‐reports more closely linked to cognitive and physical traits*” seems premature. For instance, Pergola et al. (2019) reported a significant association between polygenic risk for schizophrenia (SCZ) and peer victimization severity in a sample of 625 participants tested between 12.4 and 14.6 years of age, whereas the association was not significant before 12 years of age. This study assessed peer victimization between 12.4 and 14.6 years with peer reports, which mediated the relationship between polygenic risk and psychotic symptoms between 14.8 and 18.3 years. These aspects of the study suggested that genetic risk for SCZ may elicit adverse social reactions by other individuals during this age stage, hence encouraging an interpretation of the effects as evocative rGE. Notably, this association had a larger effect size when combining peer and teacher reports, suggesting a shared component across informants other than self. These findings directly discount the interpretation that other informants do not capture genetic risk for psychiatric disorders. Another study (Schoeler et al., 2019) analyzed data from 5028 unrelated individuals recruited in ALSPAC (The Avon Longitudinal Study of Parents and Children), a longitudinal birth cohort study, averaging self‐reports of peer victimization exposures collected at 8, 10, and 13 years of age. The main outcome of the study was the presence of an association between self‐reported peer victimization and genetic risk for depression, attention‐deficit/hyperactivity disorder (ADHD), risk‐taking behaviors, BMI, and intelligence, with a nominally significant effect of polygenic risk for SCZ. This study followed a previous report on 3988 of the same study's participants using only the 8 and 10 years‐related time points (Riglin et al., 2019). In this previous study, the authors investigated whether SCZ‐related PGS might interact with peer victimization exposure to contribute to a developmental trajectory of increasing emotional problems. They found that greater frequency and severity of peer victimization were associated with emotional problems during childhood, in turn, reinforced by peer victimization exposure, but were not directly associated with SCZ‐PGS (Riglin et al., 2019). Indeed, SCZ‐PGS correlated with childhood‐onset emotional problems but did not directly explain either the peer victimization exposure during childhood, or the trajectory of emotional problems between childhood and adolescence. Taken together, the two studies on the ALSPAC cohort suggest the possibility of a weak evocative rGE based on self‐reports, likely driven by data at the 13 years old time point. This conclusion is in line with the results found by Pergola et al., which showed an association between SCZ‐PGS and peer victimization in adolescents, but not in late infancy/puberty (10–12.5 years). These studies suggest that the identification of potential evocative rGE may vary according to age, being strongest in adolescence, while using different reports (self in ALSPAC, others in TRAILS). Once again, these data de‐emphasize the dichotomy by which different informant reports would capture different types of genetic predisposition put forward by Armitage et al. (2022), and we contend that measures of reaction (i.e., other reports) rather than a self‐assessment seem ideal to capture evocative rGE. In this kind of rGE genetic predisposition may elicit dysfunctional environmental responses that could contribute to a developmental trajectory of psychopathology manifestations. Nevertheless, the literature reviewed above suggests that differences between informants are a matter of degree more than of essence, considering that well‐powered self‐report studies targeting the appropriate age range yield results consistent with other‐informant reports.

We hope this brief discussion clarifies that many intertwined phenomena entangle pinning out discrepancies to specific latent processes; more caution is warranted. In conclusion, we do not find valid a principled distinction between self‐ and other‐reports in capturing mental health‐related aspects, except for the better interpretability of findings linking other‐reports with evocative rGE. Peer reports may be particularly relevant for the investigation of evocative rGE related to the risk for peer victimization, providing the opportunity to better understand the relationship between genetic risk and psychopathological outcomes in order to develop more accurate interventions and to prevent psychopathology. Moreover, the employment of teacher‐ and peer‐ reports in behavioral genetics research may provide insights into gene‐environment interplays related to peer victimization also during childhood, a stage in which some authors have considered peer reports more reliable than self‐reports in capturing negative social experiences (Woolway et al., 2022). In light of these considerations, the conclusion by Armitage et al. (2022) that self‐reports of peer victimization might more appropriately capture genetic liability for wellbeing requires more experimental evidence.

A word of caution is also necessary regarding the methodological outline of the study, which is based on linear regressions. Linear regression assumes that the rate of change within the association between a predictor and an outcome variable is constant—and thus linear—both within‐ and across‐ time points. However, this might not be the case for peer victimization. In the study by Pergola et al. (2019), adolescents with higher SCZ‐PGS had greater peer victimization compared to medium SCZ‐PGS adolescents, as well as with a merged sample of low/medium risk individuals. Interestingly, no differences were found between individuals with medium and lower SCZ‐PGS, suggesting non‐linearities in this gene‐environment association (e.g., the rGE may be conditional upon high risk for SCZ) which are worth investigating in future studies.

In summary, we agree with Armitage et al. (2022) that peer victimization is a complex phenomenon, with genetic and environmental influences contributing to the risk of chronic peer victimization. However, at variance with their interpretation, we highlighted that different informants might all—and not only self reports—characterize peer victimization for its associations with heritable components of wellbeing and psychiatric risk. As prior literature shows, reports from other informants may be a reliable tool for capturing how the genetic architecture of complex traits is associated with environmental factors, as they allow researchers to disambiguate at least in part between different types of GE interplay (e.g., rGE, evocative rGE) involved in this social dynamic.

## AUTHOR CONTRIBUTIONS

**Annalisa Lella**: Writing – original draft. **Linda A. Antonucci**: Supervision; Writing – review & editing. **Giulio Pergola**: Conceptualization; Funding acquisition; Supervision; Writing – review & editing.

## CONFLICT OF INTEREST

The authors have declared that they have no competing or potential conflicts of interest.

## Data Availability

Data sharing is not applicable to this article as no new data were created or analyzed in this study.
